# Regorafenib Regulates AD Pathology, Neuroinflammation, and Dendritic Spinogenesis in Cells and a Mouse Model of AD

**DOI:** 10.3390/cells9071655

**Published:** 2020-07-09

**Authors:** Kyung-Min Han, Ri Jin Kang, Hyongjun Jeon, Hyun-ju Lee, Ji-Soo Lee, HyunHee Park, Seong Gak Jeon, Kyoungho Suk, Jinsoo Seo, Hyang-Sook Hoe

**Affiliations:** 1 Department of Neural Development and Disease, Korea Brain Research Institute (KBRI), 61, Cheomdan-ro, Dong-gu, Daegu 41068, Korea; hkm5344@kbri.re.kr (K.-M.H.); flwls2001@kbri.re.kr (R.J.K.); Newace@kbri.re.kr (H.J.); hjlee@kbri.re.kr (H.-j.L.); su943c@kbri.re.kr (J.-S.L.); hyunhee16hh@gmail.com (H.P.); jsg7394@kbri.re.kr (S.G.J.); 2 Department of Brain and Cognitive Sciences, Daegu Gyeongbuk Institute of Science & Technology, Daegu 42988, Korea; 3 Department of Pharmacology, Brain Science & Engineering Institute, School of Medicine, Kyungpook National University, Daegu 41944, Korea; ksuk@knu.ac.kr

**Keywords:** regorafenib, neuroinflammation, dendritic spine, amyloid beta, tau, aging

## Abstract

The oral multi-target kinase inhibitor regorafenib, which targets the oncogenic receptor tyrosine kinase (RTK), is an effective therapeutic for patients with advanced gastrointestinal stromal tumors or metastatic colorectal cancer. However, whether regorafenib treatment has beneficial effects on neuroinflammation and Alzheimer’s disease (AD) pathology has not been carefully addressed. Here, we report the regulatory function of regorafenib in neuroinflammatory responses and AD-related pathology in vitro and in vivo. Regorafenib affected AKT signaling to attenuate lipopolysaccharide (LPS)-mediated expression of proinflammatory cytokines in BV2 microglial cells and primary cultured microglia and astrocytes. In addition, regorafenib suppressed LPS-induced neuroinflammatory responses in LPS-injected wild-type mice. In 5x FAD mice (a mouse model of AD), regorafenib ameliorated AD pathology, as evidenced by increased dendritic spine density and decreased Aβ plaque levels, by modulating APP processing and APP processing-associated proteins. Furthermore, regorafenib-injected 5x FAD mice displayed significantly reduced tau phosphorylation at T212 and S214 (AT100) due to the downregulation of glycogen synthase kinase-3 beta (GSK3β) activity. Taken together, our results indicate that regorafenib has beneficial effects on neuroinflammation, AD pathology, and dendritic spine formation in vitro and in vivo.

## 1. Introduction

Alzheimer’s disease (AD) is the most common cause of dementia among the elderly and leads to irreversible and progressive neurodegeneration, cognitive dysfunction, and eventually an impaired ability to perform simple tasks of daily life [1]. AD pathology is associated with amyloid beta (Aβ), tau protein, and neuroinflammation. Amyloid beta (Aβ), which is produced by the cleavage of the amyloid precursor protein (APP) by β- and γ-secretase, is the main component of the amyloid plaques observed in the brains of Alzheimer’s patients. Oligomerization of Aβ and its aggregation into fibrils are important for AD pathogenesis [2]. Tau is another hallmark of AD that is present in neurons but is also expressed at very low levels in central nervous system (CNS) astrocytes and oligodendrocytes [3]. Tau is abnormally phosphorylated (hyperphosphorylated) in the brains of AD patients compared to individuals without dementia. Aggregates of hyperphosphorylated tau induced by the hyperactivation of tau kinases and decreased phosphatase activity form neurofibrillary tangles (NFTs), which are typically present in the brains of AD patients [4]. Several recent studies have demonstrated that Aβ and tau cause cell toxicity and lead to synaptic and cognitive impairments in the brain [5]. Therefore, the regulation of Aβ and tau may be a useful therapeutic strategy for the prevention/treatment of AD.

The human brain comprises many different types of cells, including microglia, astrocytes, and neurons [6]. In the CNS, microglia and astrocytes have important roles in the immune response and in the defense against proinflammatory stimuli [7]. Abnormal activation of these glial cells causes neuroinflammation via the release of various proinflammatory cytokines, including interleukin-1β (IL-1β), interleukin-6 (IL-6), and inducible nitric oxide synthase (iNOS) [8]. Chronic neuroinflammation can eventually culminate in neuronal cell death and synaptic/cognitive dysfunction [9]. Thus, the elucidation of the regulatory mechanisms of glial activation may reveal potential therapeutic strategies for treating neurodegenerative diseases.

Regorafenib is a bioavailable small molecule with potential antineoplastic and antiangiogenic activities [10]. Regorafenib inhibits vascular endothelial growth factor receptors (VEGFRs) to reduce the degree of islet cell inflammation and the mutant oncogenic kinases KIT, B-RAF, and RET to inhibit tumor angiogenesis and tumor cell proliferation [10]. Multiple studies have described the beneficial effects of regorafenib on various cancers, including colorectal cancer and liver cancer, by inhibiting growth factors and angiogenesis [11,12]. However, whether regorafenib also influences neuroinflammation and AD pathogenesis associated with Aβ aggregation and tau hyperphosphorylation has not been well-studied.

In the present study, we investigated the effects of regorafenib on LPS-induced neuroinflammatory responses in BV2 microglial cells, primary microglial cells, and primary astrocytes and found that regorafenib inhibited AKT/STAT3 signaling to alter LPS-induced expression of proinflammatory cytokines. In addition, regorafenib significantly reduced gliosis in LPS-induced wild-type mice and promoted dendritic spine formation in primary hippocampal neurons, as well as in the hippocampus of 5x FAD mice brain. Finally, regorafenib-injected 5x FAD mice displayed significantly suppressed Aβ plaque levels and tau phosphorylation. These data suggest that regorafenib might be a novel therapeutic agent for AD and neuroinflammation-related diseases.

## 2. Experimental Procedures

### 2.1. Ethics Statement

All experiments were approved by the institutional biosafety committee (IBC) of the Korea Brain Research Institute (KBRI, approved no. 2014-479).

### 2.2. Cell Culture

BV2 microglial cells (a generous gift from Dr. Kyung-Ho Suk) were cultured in high-glucose Dulbecco’s Modified Eagle’s medium (DMEM) (Invitrogen, Carlsbad, CA, USA) supplemented with 5% fetal bovine serum (FBS) (Invitrogen) at 37 °C in a 5% CO_2_ incubator. APP-H4 cells (H4 cells overexpressing human APP, a generous gift from Dr. Young ho Koh) were cultured in high-glucose DMEM supplemented with gentamycin and 10% FBS at 37 °C in a 5% CO_2_ incubator.

### 2.3. Cell Viability Assay

Cell viability was determined by the 3-(4,5-dimetylthiazol-2-yl)-2,5-diphenyltetrazoliumbriomide (MTT; VWR chemicals, Solon, OH, USA) assay. The MTT assay was used to assess cell viability. In brief, BV2 microglial cells in 96-well plates were treated with regorafenib (Tokyo Chemical Industry, Tokyo, Japan; Cat No R0142) (0.1, 1, 5, 10, 20 µM) for 24 h without FBS. After adding 0.5 mg/mL MTT, the cells were further incubated for 3 h at 37 °C in a 5% CO_2_ incubator. Absorbance was measured at 580 nm.

### 2.4. mRNA Quantification by RT-PCR

RNA extracted using TRIzol (Invitrogen) according to the manufacturer’s instructions was reverse transcribed into cDNA using the Superscript cDNA Premix Kit II with oligo (dT) primers (GeNetBio, Korea). To perform RT-PCR, Prime Taq Premix (GeNetBio, Daejeon, Korea) and primers for IL-1β, IL-6, IL-18, COX-2, iNOS, TNF-α, and GAPDH were used as previously reported. After the separation of the RT-PCR products by 1.5% agarose gel electrophoresis with Eco Dye (BioFACT, Korea), images were analyzed using the ImageJ and Fusion software version 16.12 (Vilber, Collegien, France).

### 2.5. Real-Time PCR (q-PCR)

Total RNA was extracted as described above, and real-time PCR was conducted by using Fast SYBR Green Master Mix (Thermo Fisher Scientific, Waltham, MA, USA) and a QuantStudio 5 Real-Time PCR System (Thermo Fisher Scientific). The cycle threshold (Ct) value for *Gapdh* was used to normalize the Ct values of inflammatory mediator mRNAs, and the data were quantified as the fold change compared to the control.

### 2.6. Enzyme-Linked Immunosorbent Assay (ELISA)

To assess whether regorafenib affects proinflammatory cytokine levels, BV2 microglial cells were treated with LPS (200 ng/mL) or PBS for 30 min, followed by treatment of regorafenib (5 µM) or vehicle (1% DMSO) for 23.5 h. After 24 h, the conditioned media were collected, and the levels of IL-6, TNF-α (Invitrogen, Cat. No. 88-7064-88 and 88-7324-88, respectively), and IL-1β (R&D Systems, Minneapolis, MN, USA; Cat. No. Dy401) were measured using ELISA kits in accordance with the manufacturers’ instructions.

### 2.7. Antibodies and Inhibitors

Western blotting (WB), immunocytochemistry (ICC), and immunohistochemistry (IHC) were performed with the following primary antibodies: rat anti-mouse CD11b (1:400 for ICC, Abcam, Cambridge, UK), rabbit anti-COX-2 (1:200 for ICC, Abcam), rabbit anti-GFAP (1:500 for ICC, Wako, Japan), rabbit anti-Iba-1 (1:500 for ICC, Wako, Osaka, Japan), rabbit anti-AKT (1:1000 for WB, Santa Cruz Biotechnology, Santa Cruz, CA, USA), rabbit anti-p-AKT (Ser473) (1:1000 for WB, Cell Signaling Technology, Danvers, MA, USA), rabbit anti-STAT3 (1:1000 for WB, Cell Signaling Technology), rabbit anti-p-STAT3 (Ser727, 1:1000 for WB, 1:200 for ICC, Abcam), rabbit anti-p-STAT3 (Y705, 1:1000 for WB, 1:200 for ICC, Cell Signaling Technology), rabbit anti-P38 (1:1000 for WB, Cell Signaling Technology), rabbit anti-p-P38 (1:1000 for WB, Cell Signaling Technology), mouse anti-β-actin (1:1000 for WB, Santa Cruz Biotechnology), mouse anti-6E10 (1:1000 for WB, Biolegend, San Diego, CA, USA), mouse anti-BACE1 (1:200 for IHC, Abcam), mouse anti-4G8 (1:200 for IHC, Biolegend), mouse anti-ATG5 (1:200 for IHC, Novus, Centennial, CO, USA), mouse anti-ATG12 (1:200 for IHC, R&D Systems), rabbit anti-p-GSK3β (Y216) (1:200 for IHC, Abcam), rabbit anti-p-CDK5 (1:200 for IHC, Abacm), rabbit anti-DYRK1A (1:200 for IHC, Abcam), rabbit anti-NEP (1:200 for IHC, Abcam), and rabbit anti-ADAM17 (1:200 for IHC, Abcam). To inhibit TLR4 and AKT, TAK-242 (500 nM, Calbiochem, La Jolla, CA, USA) and MK2206 (10 µM, Selleckchem, Houston, TX, USA) were used, respectively. LPS was from *Escherichia coli* O111:B4 (Sigma-Aldrich, St. Louis, MO, USA).

### 2.8. Western Blotting

Western blot analyses were performed in BV2 microglial cells, primary microglia, and astrocytes as previously described [13]. Fusion or ImageJ software was used to analyze blot images.

### 2.9. Immunocytochemistry

To examine dendritic spine formation, primary hippocampal neurons were cultured from Sprague-Dawley rat embryos, as previously described [14]. After transfection with GFP plasmid DNA (Addgene, Watertown, MA, USA; Cat. No. 6085-1), primary hippocampal neurons were treated with regorafenib or vehicle for 24 h before immunocytochemistry.

BV2 microglia were fixed with 4% paraformaldehyde for 10 min, washed three times with PBS, and incubated overnight with primary antibodies in GDB buffer consisting of 0.1% gelatin, 0.3% Triton X-100, 16 mM sodium phosphate, pH 7.4, and 450 mM NaCl at 4 °C. The cells were subsequently washed three times with PBS and incubated with fluorescent dye-conjugated secondary antibodies (Molecular Probes, Eugene, OR, USA) for 1 h at room temperature. The cells were mounted in DAPI-containing solution (Vector Laboratories, Burlingame, CA, USA). Th images were captured from a single plane using a confocal microscope (Nikon A1, Nikon, Tokyo, Japan) and analyzed using the ImageJ software.

### 2.10. Cytosolic and Nuclear Fractionation

Cells were lysed in cytosol fractionation buffer composed of (mM) 10 HEPES pH 8.0, 1.5 MgCl_2_, 10 KCl, 0.5 DTT, 300 sucrose, and 0.5 PMSF and 0.1% NP-40 for 5 min. After centrifugation of the lysates at 10,000 rpm for 1 min at 4 °C, the supernatant was stored as the cytosolic fraction, and the pellet was further lysed on ice for 15 min in nuclear fractionation buffer composed of (mM) 10 HEPES pH 8.0, 100 KCl, 100 NaCl, 0.2 EDTA, 0.5 DTT, and 0.5 PMSF and 20% glycerol before centrifugation for 15 min at 10,000 rpm and 4 °C.

### 2.11. Primary Microglial Cell and Astrocyte Culture

Primary microglia and astrocytes were prepared as previously described [15]. Briefly, whole brains of postnatal one-day-old C57BL/6 mice were mechanically disrupted using a 70 μm nylon mesh. The mixed glial cells were seeded in T75 flasks in low-glucose DMEM supplemented with 10% FBS and penicillin-streptomycin. To isolate primary astrocytes, the mixed glial cells were shaken at 250 rpm overnight, and the adherent cells were retained and dissociated in trypsin-EDTA before centrifugation (1200 rpm, 30 min). After removing the conditioned medium, the cell pellet containing primary astrocytes was retained. To isolate primary microglial cells, the mixed glial cells were subjected to mild trypsin digestion (five-fold dilution of 0.25% trypsin in serum-free DMEM) for 40 min in a 5% CO_2_ incubator at 37 °C. After discarding the upper astrocyte layer, low-glucose DMEM containing 10% FBS was added, and the remaining primary microglial cells were used in experiments.

### 2.12. Animals

Male C57BL6/N mice were purchased from Orient-Bio (Seongnam, Korea) and housed in a pathogen-free facility with 12 h of light and dark per day. F1 generation 5x FAD mice were purchased from the Jackson Laboratory. All experiments were performed in accordance with approved animal protocols and guidelines established by the Korea Brain Research Institute (IACUC-2016-0013).

### 2.13. Immunohistochemistry

Mice were perfused and fixed with 4% paraformaldehyde (PFA). Brain tissues were flash-frozen, sliced (35 µm thick) using a cryostat, rinsed with PBS, and permeabilized for 1 h in PBS containing 0.2% Triton X-100 and 1% BSA. The tissue sections were subsequently incubated with primary antibodies at 4 °C overnight. After washing three times with PBS containing 0.5% BSA, the sections were incubated with fluorescent dye-conjugated secondary antibodies for 1 h at room temperature.

### 2.14. Golgi Staining

To test the effects of regorafenib on dendritic spinogenesis in vivo, we conducted Golgi staining using an FD Rapid GolgiStain Kit (FD Neurotechnologies Inc., Ellicott City, MD, USA) as described previously [16]. Briefly, regorafenib- or vehicle-injected animals were submerged in Solutions A and B for two weeks in the dark and transferred to Solution C for 24 h. Solution C was replaced after the first 24 h, and individual mouse brains were sliced at a 150 µm thickness using a VT1000S Vibratome (Leica, Wetzlar, Germany). Dendritic images were acquired by an Axioplan 2 (Zeiss, Oberkochen, Germany) under bright-field microscopy. To measure the dendritic spine density, we used 8–10 slices of each mouse brain from −1.70 mm to −2.30 mm relative to the bregma. The ImageJ software was used for data analysis.

### 2.15. Statistical Analyses

GraphPad Prism 6 software (GraphPad Software Inc., La Jolla, CA, USA) was used for data analysis. The unpaired two-tailed Student’s *t*-test with Welch’s correction or one-way ANOVA was used for comparisons of the two groups or multiple comparisons, respectively. Tukey’s test was conducted for posthoc analysis (* *p* < 0.05, ** *p* < 0.01, *** *p* < 0.0001). Data are presented as the mean ± S.E.M.

## 3. Results

### 3.1. Regorafenib Regulates LPS-Induced Expression of Proinflammatory Cytokines

**Figure 1:**
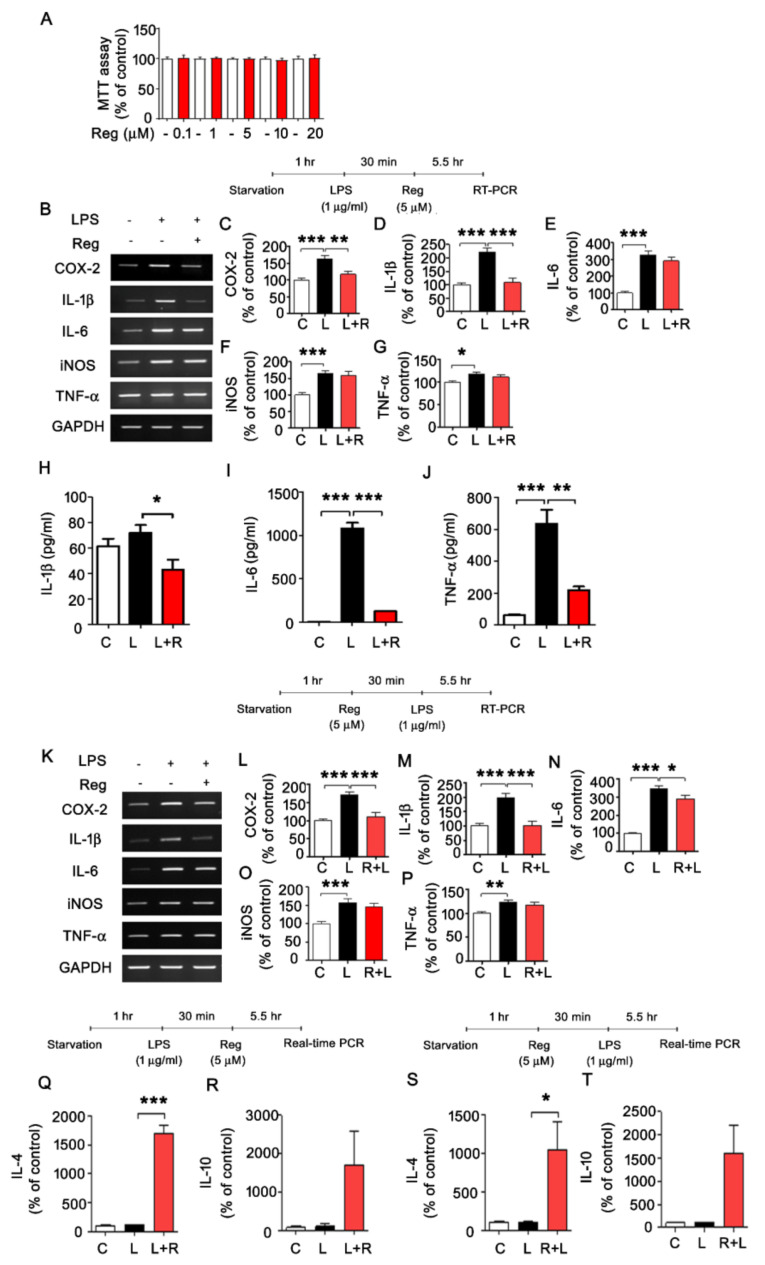
LPS-induced proinflammatory and anti-inflammatory cytokine levels are altered in regorafenib-treated BV2 microglial cells. (**A**) MTT assays were performed after treating BV2 microglial cells with regorafenib (0.1, 1, 5, 10, 20 µM) or vehicle (1% DMSO) (n = 18/group). (**B**–**G**) BV2 microglial cells were pretreated with LPS (1 µg/mL) or PBS for 30 min, followed by treatment with regorafenib (5 µM) or vehicle (1% DMSO) for 5.5 h and measurement of proinflammatory cytokine levels (n = 12/group). (**H**–**J**) BV2 microglial cells were pretreated with LPS (200 ng/mL) or PBS for 30 min, followed by treatment with regorafenib (5 µM) or vehicle (1% DMSO) for 23.5 h and measurement of proinflammatory cytokine levels using ELISA (n = 4/group). (**K**–**P**) BV2 microglial cells were pretreated with regorafenib (5 µM) or vehicle (1% DMSO) for 30 min, followed by treatment with LPS (1 µg/mL) or PBS for 5.5 h and measurement of proinflammatory cytokine levels (n= 12/group). (**Q**–**R**) BV2 microglial cells were pretreated with LPS (1 µg/mL) or PBS for 30 min, followed by treatment with regorafenib (5 µM) or vehicle (1% DMSO) for 5.5 h. Then, total RNA was isolated, and anti-inflammatory cytokine levels were measured by real-time PCR (IL-4, IL-10; n = 3/group). (**S**–**T**) BV2 microglial cells were pretreated with regorafenib (5 µM) or vehicle (1% DMSO) for 30 min, followed by treatment with LPS (1 µg/mL) or PBS for 5.5 h. Then, total RNA was isolated, and anti-inflammatory cytokine levels were measured by real-time PCR (IL-4, IL-10; n = 3/group). * *p* < 0.05, ** *p* < 0.01, *** *p* < 0.001.

The RTK inhibitor regorafenib is currently used for cancer treatment, but whether regorafenib affects LPS-induced neuroinflammation and its underlying mechanism has not been established. To address this point, we first tested whether regorafenib has cytotoxicity in BV2 microglial cells. MTT assays of BV2 microglial cells treated with regorafenib (up to 20 μM) for 24 h revealed no effect of regorafenib on the cytotoxicity of BV2 microglial cells (Figure 1A).

To determine the effects of regorafenib on LPS-induced proinflammatory cytokine release, BV2 microglial cells were incubated for 30 min with LPS (1 μg/ml) or PBS before treatment with regorafenib (5 μM) or vehicle (1% DMSO) for 5.5 h. Treatment with 5 μM regorafenib significantly suppressed LPS-induced upregulation of COX-2 and IL-1β mRNA levels but not IL-6, iNOS, and TNF-α mRNA levels (Figure 1B–G). In addition, when we treated BV2 cells with LPS for 6 h, we observed that it was usually a 1.5–2-fold increase in mRNA expression of proinflammatory cytokines; COX-2, IL-1β, IL-6, and iNOS (but not TNF-α compared to cells treated with vehicle.

We then investigated whether regorafenib could differentially affect LPS-evoked proinflammatory cytokine secretion by ELISA. BV2 microglial cells were incubated for 30 min with LPS or PBS before treatment with regorafenib or vehicle for 23.5 h. Importantly, we found that regorafenib significantly attenuated LPS-induced increases in the secretion of IL-1β, IL-6, and TNF-α (Figure 1H–J).

Next, we examined the preventative effects of regorafenib on the LPS-mediated proinflammatory response by pretreating BV2 microglial cells for 30 min with regorafenib or vehicle for 30 min before treatment with LPS or PBS for 5.5 h. Regorafenib pretreatment significantly attenuated LPS-mediated induction of COX-2, IL-1β, and IL-6 transcripts (Figure 1K–P).

To test whether regorafenib could alter anti-inflammatory responses, BV2 microglia were incubated for 30 min with LPS or PBS before treatment with regorafenib or vehicle for 5.5 h. Regorafenib significantly increased IL-4 mRNA levels in BV2 microglia pretreated with LPS (Figure 1Q–R). In addition, pretreatment with regorafenib also significantly upregulated IL-4 mRNA levels in cells treated with LPS (Figure 1S–T). These data suggest that regorafenib regulates the LPS-induced inflammatory response by reducing proinflammatory cytokines and increasing anti-inflammatory cytokines in BV2 microglial cells.

### 3.2. Regorafenib Modulates AKT/STAT3 Signaling to Alter LPS-Induced Neuroinflammatory Responses

**Figure 2:**
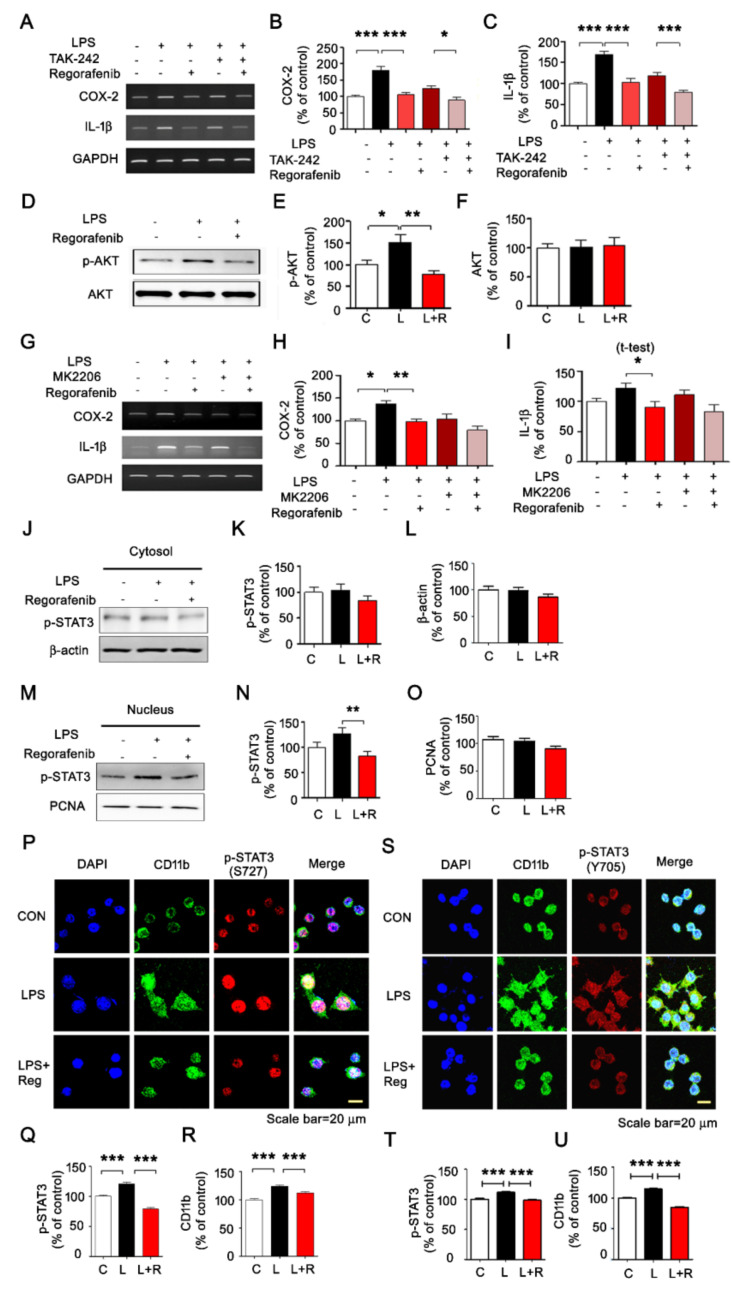
The effects of regorafenib on LPS-induced proinflammatory cytokine levels are dependent on TLR4/AKT signaling. (**A**) BV2 microglial cells were pretreated with LPS (1 µg/mL) or PBS for 30 min, treated with TAK-242 (TLR4 inhibitor, 500 nM) or vehicle (1% DMSO) for 30 min, followed by sequential treatment with regorafenib (5 µM) or vehicle (1% DMSO) for 5 h. Then, total RNA was isolated, and IL-1β or COX-2 mRNA levels were measured by RT-PCR. (**B**,**C**) Quantification of data from **A** (IL-1β or COX-2: Veh, n = 18; LPS, n = 18; regorafenib + LPS, n = 18; TAK-242 + LPS, n = 18; TAK-242 + regorafenib + LPS, n = 18). (**D**–**F**) BV2 microglial cells were pretreated with LPS (1 μg/ml) or PBS for 45 min, followed by treatment with regorafenib (5 μM) or vehicle (1% DMSO) for 45 min and western blotting with anti-p-AKT or anti-AKT antibodies (n = 6/group). (**G**–**I**) BV2 microglial cells were pretreated with LPS (1 μg/ml) or PBS for 30 min, followed by treatment with MK2206 (AKT inhibitor, 10 μM) or vehicle (1% DMSO) for 30 min. These cells were further treated with regorafenib (5 μM) or vehicle (1% DMSO) for 5 h, and proinflammatory cytokine levels were measured (n = 7/group). (**J**–**O**) BV2 microglial cells were pretreated with LPS (1 μg/ml) or PBS for 30 min, followed by treatment with regorafenib (5 μM) or vehicle (1% DMSO) for 5.5 h and subcellular fractionation (n = 27/group). (**P**–**U**) BV2 microglial cells were pretreated with LPS (1 μg/ml) or PBS for 30 min, followed by treatment with regorafenib (5 μM) or vehicle (1% DMSO) for 5.5 h and immunocytochemistry with anti-p-STAT3(Ser^727^) (Veh, n = 643; LPS, n = 614; regorafenib + LPS, n = 577) and anti-CD11b (Veh, n = 712; LPS, n = 666; regorafenib + LPS, n = 536) antibodies or anti- p-STAT3(Y^705^) (Veh, n = 410; LPS, n = 462; regorafenib + LPS, n = 554) and anti-CD11b antibodies (Veh, n = 426; LPS, n = 539; regorafenib + LPS, n = 656). * *p* < 0.05, ** *p* < 0.01, *** *p* < 0.001.

To elucidate the underlying mechanisms by which regorafenib alters LPS-induced proinflammatory responses, we inspected signaling pathways linked to toll-like receptor 4 (TLR4), which recognizes LPS. For these experiments, BV2 microglial cells were preincubated for 30 min with LPS or PBS before successive treatment with a TLR4 inhibitor (TAK-242, 500 nM) or vehicle for 30 min and regorafenib or vehicle for 5 h. Regorafenib significantly reduced LPS-induced upregulation of COX-2 and IL-1β transcripts (Figure 2A–C). Interestingly, regorafenib further decreased LPS-induced COX-2 and IL-1β mRNA levels in BV2 microglia pretreated with TAK-242, although TAK-242 pretreatment did not have any additive effects on LPS-induced expression of proinflammatory cytokines in regorafenib-treated BV2 microglia (Figure 2A–C). These results indicate that the regulatory function of regorafenib in the LPS-induced proinflammatory response is partially dependent on TLR4 signaling.

To determine whether the LPS-induced AKT signaling pathway is regulated by regorafenib, BV2 microglia were incubated successively with LPS or PBS for 45 min and regorafenib or vehicle for 45 min. Regorafenib-treated BV2 microglial cells showed a significant reduction of LPS-induced p-AKT upregulation, while the total levels of AKT were not altered (Figure 2D–F).

We then tested whether regorafenib alters LPS-mediated expression of proinflammatory cytokines in an AKT-dependent manner. BV2 microglia were preincubated for 30 min with LPS or PBS before successive treatment with an AKT inhibitor (MK2206, 10 μM) or vehicle for 30 min and regorafenib or vehicle for 5 h. Treatment with MK2206, LPS, and regorafenib did not alter LPS-induced upregulation of IL-1β or COX-2 mRNA compared to treatment with LPS and regorafenib or MK2206 and LPS (Figure 2G–I). These data suggest that regorafenib inhibits AKT signaling to alter LPS-induced expression of proinflammatory cytokines.

To investigate whether regorafenib also regulates JAK/STAT signaling pathways, we performed subcellular fractionation to measure levels of p-STAT3 in the cytosol and nucleus in LPS-treated BV2 microglial cells. Regorafenib significantly downregulated nuclear p-STAT3 (Ser^727^) levels without affecting cytosolic p-STAT3 (Ser^727^) levels (Figure 2J-O). Immunocytochemistry with antibodies against p-STAT3 (Ser^727^) and CD11b, or p-STAT3 (Tyr^705^) and CD11b also showed that regorafenib significantly reduced levels of nuclear p-STAT3 and CD11b in BV2 microglia (Figure 2P–U).

### 3.3. Regorafenib Alters LPS-Induced Expression of Proinflammatory Cytokines in Primary Microglia and Astrocytes

**Figure 3:**
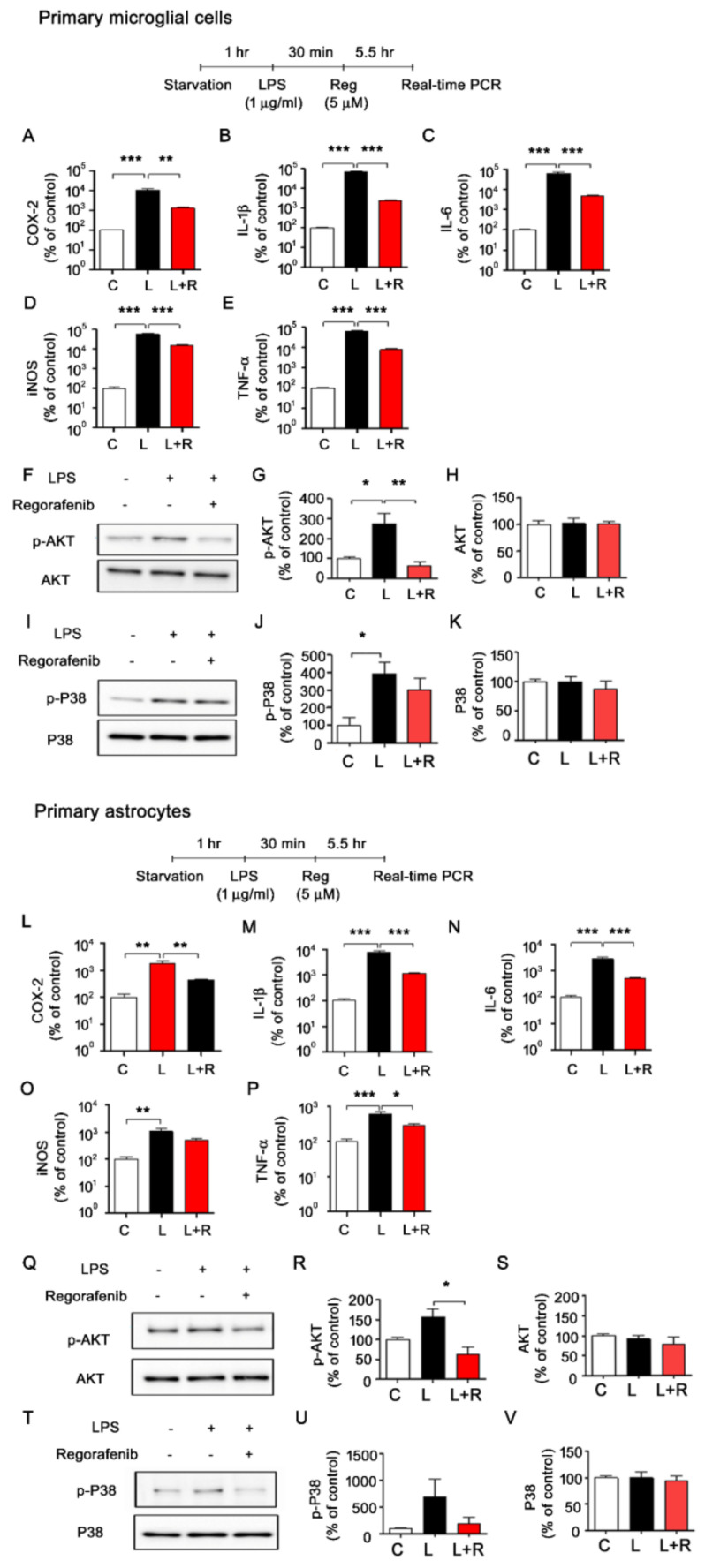
Regorafenib regulates LPS-induced proinflammatory cytokine levels in primary microglia and astrocytes. (**A**–**E**) Primary microglial cells were pretreated with LPS (1 µg/mL) or PBS for 30 min, followed by treatment with regorafenib (5 µM) or vehicle (1% DMSO) for 5.5 h and measurement of proinflammatory cytokine levels (n = 4/group). (**F**–**K**) Primary microglial cells were pretreated with LPS (1 µg/mL) or PBS for 45 min, followed by treatment with regorafenib (5 µM) or vehicle (1% DMSO) for 45 min and western blotting with anti-p-AKT, anti-AKT, anti-p-P38, or anti-P38 antibodies (n = 3/group). (**L**–**P**) Primary astrocytes were pretreated with LPS (1 µg/mL) or PBS for 30 min, followed by treatment with regorafenib (5 µM) or vehicle (1% DMSO) for 5.5 h and measurement of proinflammatory cytokine levels (n = 4/group) (**Q**–**V**) Primary astrocytes were pretreated with LPS (1 µg/mL) or PBS for 45 min, followed by treatment with regorafenib (5 µM) or vehicle (1% DMSO) for 45 min and western blotting with anti-p-AKT, anti-AKT, anti-p-P38, or anti-P38 antibodies (n = 3/group). * *p* < 0.05, ** *p* < 0.01, *** *p* < 0.001.

We first assessed the effects of regorafenib on LPS-induced neuroinflammation in primary glial cells cultured under low-glucose conditions. Consistent with the data from BV2 microglial cells, the LPS-induced upregulation of transcripts of proinflammatory cytokines was in primary microglial cells, almost abolished by regorafenib treatment (Figure 3A–E). In addition, regorafenib significantly reduced LPS-mediated p-AKT levels but not p-P38 in primary microglial cells (Figure 3F–K).

Next, we tested whether regorafenib regulated LPS-mediated neuroinflammatory responses in primary astrocytes, another type of glial cell in the brain. We found that regorafenib significantly suppressed LPS-stimulated COX-2, IL-1β, IL-6, and TNF-βmRNA levels in primary astrocytes (Figure 3L–P). iNOS mRNA levels in LPS-treated primary astrocytes showed a decreasing trend in response to regorafenib (Figure 3L–P). We further observed that regorafenib downregulated LPS-induced AKT phosphorylation but not levels of total AKT or P38 phosphorylation in primary astrocytes (Figure 3Q–V). These data showed that regorafenib attenuates microglial and astrocytic LPS-induced neuroinflammatory responses.

### 3.4. Regorafenib Attenuates LPS-Induced Glial Activation and the Expression of Proinflammatory Cytokines in Wild-Type Mice

**Figure 4:**
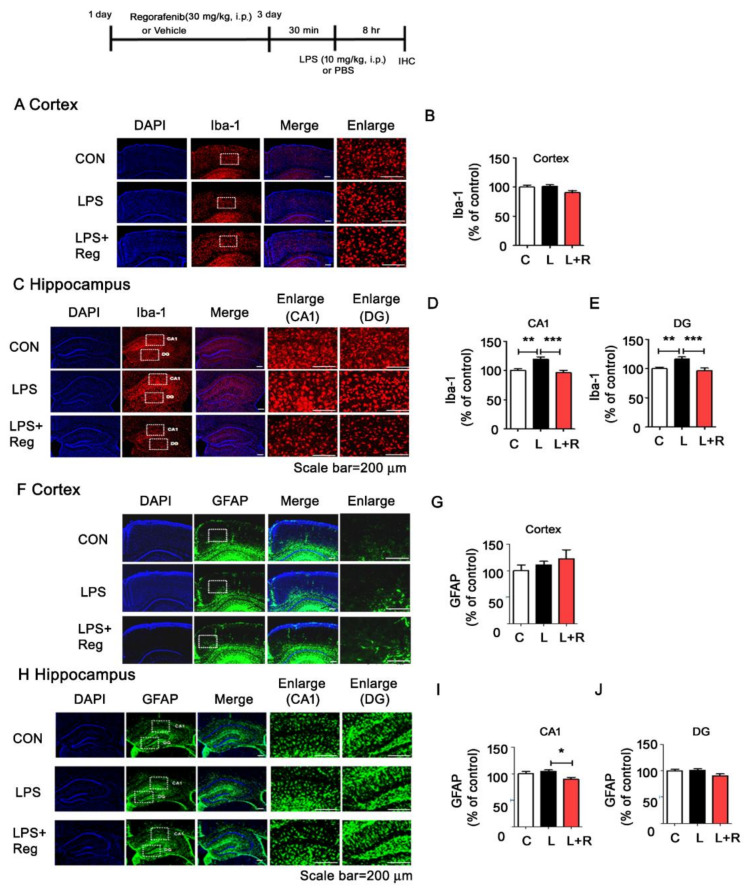
Regorafenib modulates LPS-induced microglial and astrocyte activation as well as proinflammatory cytokine upregulation in vivo. (**A**–**E**) Wild-type mice were injected with regorafenib (30 mg/kg, i.p.) or vehicle (2% DMSO + 30 % PEG + 5 % Tween 80) daily for three days and subsequently treated with LPS (10 mg/kg, i.p.) or PBS for 8 h. Then, the treated wild-type mice were perfused and fixed, and immunohistochemistry was performed with anti-Iba-1(**A**–**E**) or anti-GFAP (**F**–**J**) antibodies in the cortex and hippocampus (n = 7 mice/group). * *p* < 0.05, ** *p* < 0.01, *** *p* < 0.001.

Since we observed inhibitory effects of regorafenib on LPS-induced proinflammatory cytokine expression in BV2 microglial and primary glial cells, we then examined whether regorafenib modulated LPS-induced neuroinflammation in vivo. Wild-type mice were injected with regorafenib (30 mg/kg, intraperitoneal (i.p.)) or vehicle (2% DMSO + 30% polyethylene glycol + 5% Tween 80) daily for three days, followed by treatment with LPS (10 mg/kg, i.p.) or PBS [17,18]. After 8 h, the wild-type mice were sacrificed, and brain tissues were subjected to immunohistochemistry. Using anti-ionized calcium-binding adapter molecule-1 (Iba-1) and anti-glial fibrillary acidic protein (GFAP) antibodies, we found that regorafenib significantly reduced LPS-induced microglial activation in the hippocampus but not in the cortex (Figure 4A-E). Similarly, regorafenib decreased LPS-induced astrocyte activation in CA1 of the hippocampus but not in the cortex and hippocampal DG region (Figure 4F–J).

**Figure 5:**
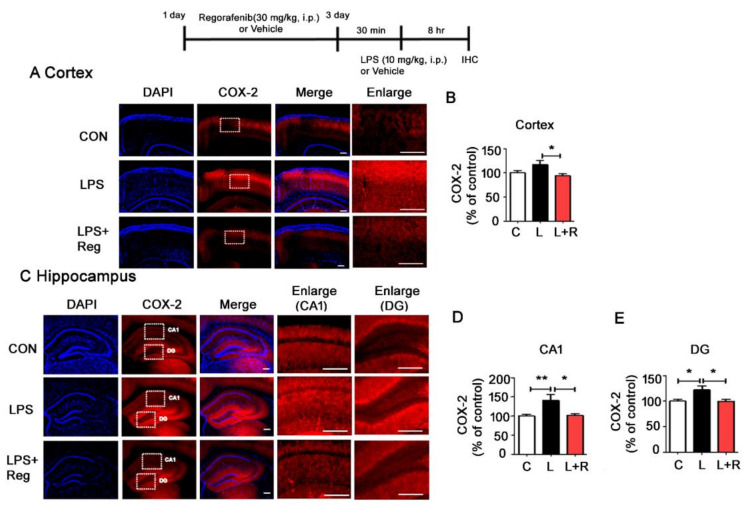
Regorafenib modulates LPS-induced proinflammatory cytokine COX-2 upregulation in vivo. (**A**–**E**) Wild-type mice were injected with regorafenib (30 mg/kg, i.p.) or vehicle (2% DMSO + 30 % PEG + 5 % Tween 80) daily for three days and subsequently treated with LPS (10 mg/kg, i.p.) or PBS for 8 h. Then, the treated wild-type mice were perfused and fixed, and immunohistochemistry was performed with anti-COX-2 antibody (n = 7 mice/group). * *p* < 0.05, ** *p* < 0.01.

To test whether regorafenib regulates LPS-induced upregulation of proinflammatory cytokines, we examined COX-2 levels in wild-type mice injected with regorafenib or vehicle prior to treatment with LPS. Regorafenib significantly reduced LPS-derived COX-2 upregulation in both the cortex and hippocampus (Figure 5A–E).

Next, we assessed the effects of regorafenib on Aβ-mediated neuroinflammation. Three-month-old 5x FAD mice were injected with regorafenib or vehicle daily for two weeks, and immunohistochemistry was conducted with anti-Iba-1 and anti-GFAP antibodies. Unlike LPS-treated wild-type mice, regorafenib did not affect the Aβ-mediated microglial activation in 5x FAD mice (Figure S1A–E). In addition, regorafenib-injected 5x FAD mice exhibited significantly reduced Aβ-mediated astrocyte activation in the cortex but not the hippocampus (Figure S1F–J). These data indicate that regorafenib modulates LPS-induced neuroinflammatory responses but has less of an effect on Aβ-mediated neuroinflammation in vivo.

### 3.5. Regorafenib Significantly Increases Dendritic Spine Density in Primary Hippocampal Neurons and in the Brains of 5x FAD Mice

**Figure 6:**
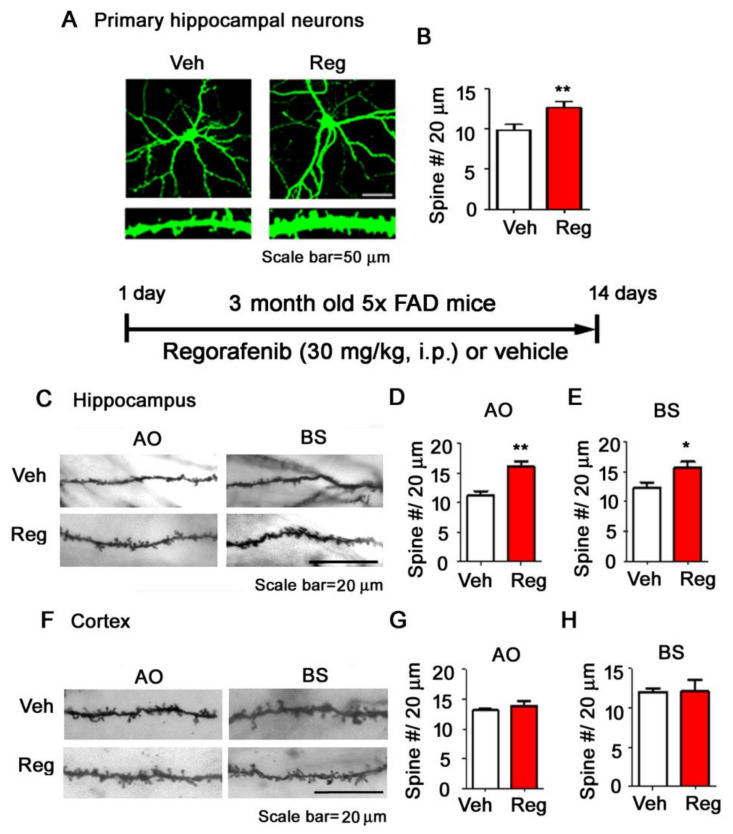
Regorafenib promotes dendritic spine formation in primary hippocampal neurons and in the brains of 5x FAD mice. (**A**–**B**) Primary hippocampal neurons were transfected with GFP and treated with regorafenib (5 µM) or vehicle (1% DMSO) for 24 h before measuring dendritic spine density (Veh, n = 20; regorafenib, n = 21). (**C**–**H**) 5x FAD mice were injected with regorafenib (30 mg/kg, i.p.) or vehicle (2% DMSO + 30 % PEG + 5 % Tween 80) daily for two weeks, and Golgi staining was conducted in the hippocampus CA1 and cortical layer V (n = 4 mice/group). * *p* < 0.05, ** *p* < 0.01.

To examine whether regorafenib can alter dendritic spine formation (which is associated with learning and memory), a GFP-expressing vector was introduced into primary hippocampal neurons by transfection to permit the visualization of dendrite spines before treatment with regorafenib or vehicle. Measurements of the number of dendritic spines 24 h after treatment revealed that regorafenib significantly increased the dendritic spine number in primary hippocampal neurons compared to vehicle treatment (Figure 6A–B).

We then addressed whether regorafenib could increase the dendritic spine number in the brains of AD mice. Importantly, 5x FAD mice injected with regorafenib daily for two weeks displayed an increased number of dendritic spines in apical oblique (AO) and basal (BS) dendrites of the hippocampus compared to vehicle-treated 5x FAD mice, although the same effect was not observed in the cortex (Figure 6C–H). Taken together, these data indicate that regorafenib could promote dendritic spine formation, and stability in primary hippocampal neurons and in the hippocampus of 5x FAD mice.

### 3.6. Regorafenib Significantly Downregulates Aβ Plaque Levels in 5x FAD Mice

**Figure 7:**
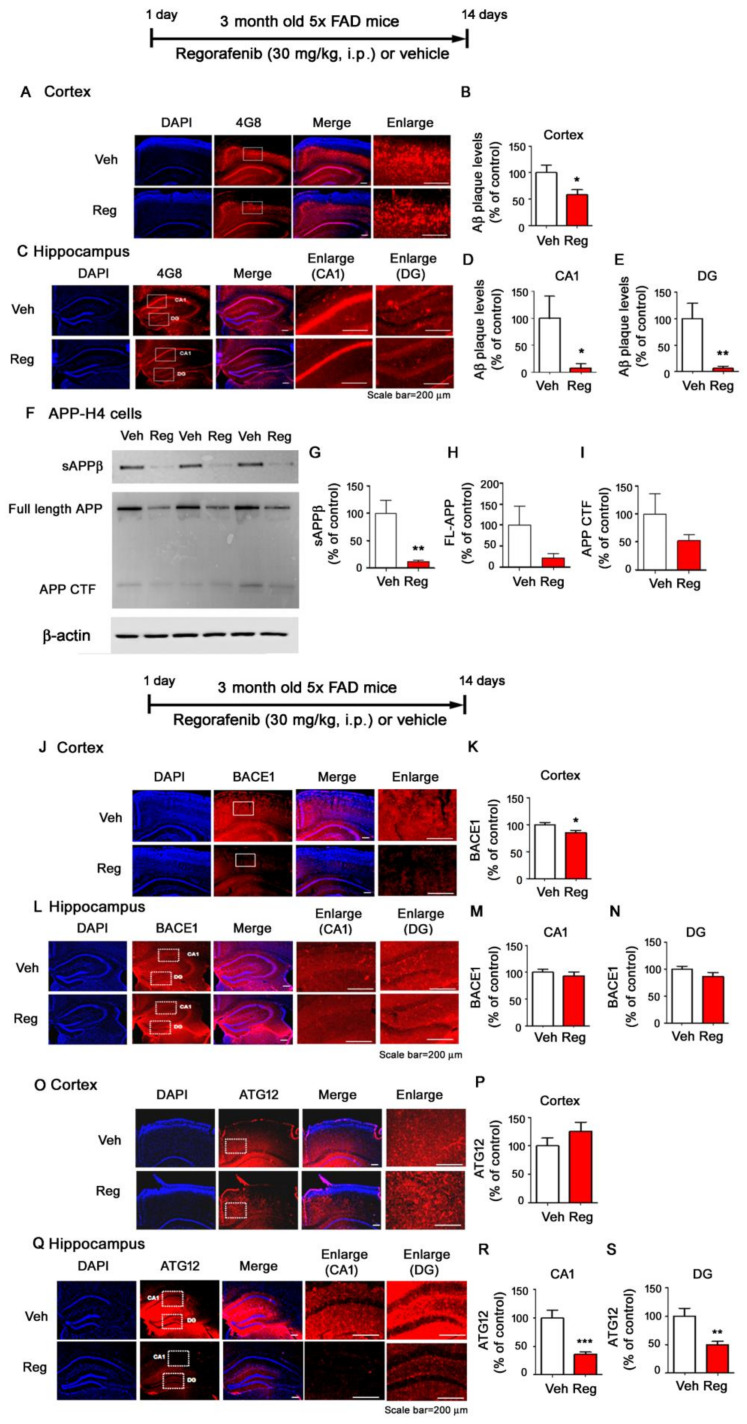
Amyloid plaque levels are significantly decreased in the brains of regorafenib-injected 5x FAD mice. (**A**–**E**) 5x FAD mice were injected with regorafenib (30 mg/kg, i.p.) or vehicle (2% DMSO + 30% PEG + 5% Tween 80) daily for two weeks, and immunohistochemistry was performed with an anti-4G8 antibody (n = 4 mice/group). (**F**–**I**) APP-H4 cells were treated with regorafenib (5 µM) or vehicle (1% DMSO) for 24 h, and western blotting was conducted to measure sAPPβ, full-length APP, and APP CTF levels (n = 6/group). (**J**–**S**) 5x FAD mice were injected with regorafenib (30 mg/kg, i.p.) or vehicle (2% DMSO + 30 % PEG + 5 % Tween 80) daily for two weeks, and immunohistochemistry was performed with anti-BACE1 (**J**–**N**, n = 4 mice/group) or anti-ATG12 antibodies in the cortex and hippocampus (**O**–**S**, n = 5 mice/group). * *p* < 0.05, ** *p* < 0.01, *** *p* < 0.001.

To determine if regorafenib had beneficial effects on Aβ pathology, we investigated Aβ plaque levels with an anti-4G8 antibody in the brains of three-month-old 5x FAD mice injected with regorafenib or vehicle daily for two weeks. Interestingly, we found that regorafenib significantly downregulated amyloid plaque deposition in both the cortex and hippocampus of 5x FAD mice compared with vehicle-treated animals (Figure 7A–E).

We then investigated whether regorafenib regulated APP processing to alter Aβ plaque levels. We treated H4 neuroglioma cells overexpressing human APP (APP-H4 cells) with regorafenib or vehicle for 24 h and measured sAPPβ, full-length APP (FL-APP), and APP C-terminal fragment (CTF) levels. We found that regorafenib-treated APP-H4 cells showed significantly reduced sAPPβ levels and a trend toward decreased FL-APP and APP CTF levels compared to vehicle-treated cells (Figure 7F–I).

Next, we tested whether regorafenib modulated α-secretase (ADAM17) and β-secretase (BACE1) levels to alter Aβ pathology. We observed that regorafenib-injected 5x FAD mice exhibited decreased BACE1 levels in the cortex but not the hippocampus (Figure 7J–N). In addition, regorafenib-injected 5x FAD mice did not alter α-secretase ADAM17 levels in the cortex and hippocampus (Figure S2A–E).

We next examined whether regorafenib could alter Aβ degradation/secretion and consequently reduce Aβ generation. We found that regorafenib did not alter the levels of neprilysin (NEP), a major Aβ-degrading enzyme, in the cortex and hippocampus in 5x FAD mice (Figure S3A–E). However, we found that regorafenib significantly downregulated the autophagy protein ATG12 in the hippocampus in 5x FAD mice, which was associated with Aβ production and secretion (Figure 7O–S). The levels of ATG5, a substrate of ATG12, were not altered by regorafenib, although there was a trend toward reductions in the cortex and hippocampus (Figure S4A–E).

### 3.7. Regorafenib Significantly Reduces Tau Phosphorylation at T212 and S214 and Tau Kinase GSK3β Activity in the Brains of 5x FAD Mice

**Figure 8:**
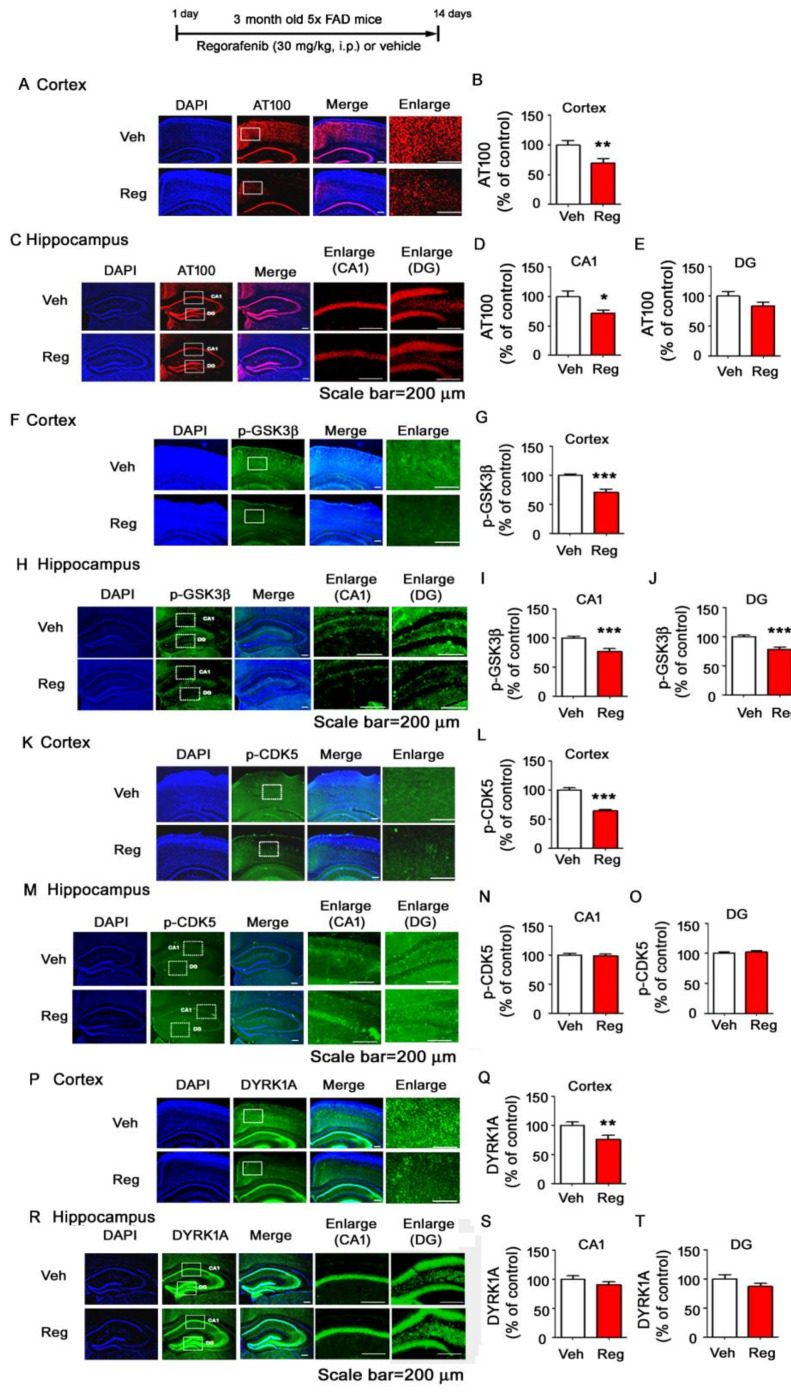
Regorafenib significantly downregulates tau phosphorylation at T212 and S214 and GSK3β activity in 5x FAD mice. (**A**–**T**) 5x FAD mice were injected with regorafenib (30 mg/kg, i.p.) or vehicle (2% DMSO + 30% PEG + 5% Tween 80) daily for two weeks, and immunohistochemistry was performed with anti-AT100 (**A**–**E**, n = 4 mice/group), anti-p-GSK3β (Y216) (**F**–**J**, n = 4 mice/group), anti-p-CDK5 (**K**–**O**, Veh, n = 3 mice/group; regorafenib, n= 4 mice/group), or anti-DYRK1A antibodies (**P**–**T**, n = 4 mice/group). * *p* < 0.05, ** *p* < 0.01, *** *p* < 0.001.

To determine the effects of regorafenib on tau phosphorylation, another hallmark of AD, three-month-old 5x FAD mice injected with regorafenib or vehicle daily for two weeks were subjected to immunohistochemistry using anti-AT8, anti-AT100, anti-AT180, or anti-Tau-5 antibodies. We observed that regorafenib prominently reduced phosphorylation of tau at T212 and S214 in the cortex and hippocampus CA1 of 5x FAD mice without affecting levels of phosphorylation at S202, T205, and T231 (Figure 8A–E, Figure S5). Additionally, regorafenib decreased total tau levels in the cortex but not the hippocampus of 5x FAD mice (Figure S6).

To elucidate the molecular mechanisms by which regorafenib alters tau phosphorylation, we measured tau kinase levels by conducting immunohistochemistry with anti-p- -GSK3β (Y216), anti-p-CDK5, or anti-DYRK1A antibodies in the brains of 5x FAD mice treated with regorafenib or vehicle. Importantly, regorafenib-injected 5x FAD mice displayed significantly decreased levels of pY216 of GSK3β in the cortex and hippocampus compared to vehicle-treated 5x FAD mice (Figure 8F–J). Additionally, regorafenib decreased p-CDK5 and DYRK1A levels in the cortex but not the hippocampus of 5x FAD mice (Figure 8K–T). These data indicate that regorafenib might induce a reduction of tau phosphorylation at T212 and S214 by inhibiting the activity of GSK3β.

## 4. Discussion

The small molecule regorafenib inhibits various kinases associated with normal cellular functions and pathological processes, such as oncogenesis and tumor angiogenesis [10]. In in vitro assays, clinically relevant concentrations of regorafenib or M-2 and M-5, the major human active metabolites of regorafenib, inhibit the activity of VEGFR1/2/3, TrkA, RAF-1, PTK5, and Abl [10]. In vivo, regorafenib displays anti-angiogenic and anti-metastatic activities and inhibits tumor growth in rodent models, including human colorectal carcinoma xenograft mice [19].

Tyrosine kinase inhibitors such as dasatinib and ibrutinib regulate LPS-induced proinflammatory cytokine levels in vitro. For instance, we and others recently demonstrated that dasatinib affects LPS-induced microglial and astrocytic proinflammatory cytokine levels [15,20]. We also reported regulatory effects of ibrutinib, a Bruton tyrosine kinase inhibitor, on LPS-induced expression of proinflammatory cytokines in BV2 microglia and primary cultured microglia [21]. The tyrosine kinases c-Abl and Src have also been implicated in regulating the LPS-mediated immune response. Le et al. found that c-Abl activity regulates the LPS-mediated proinflammatory response in macrophages [22]. Another study showed that Src modulates LPS-induced local inflammation in colorectal cancer [23]. Interestingly, several studies have demonstrated that regorafenib reduces pulmonary inflammation by decreasing M1 macrophages and increasing M2 macrophages [24] and significantly decreases secreted TNF-α, IL-1β, and IL-6 levels in SK-Hep1 cells [25]. However, whether regorafenib alters LPS-induced proinflammatory cytokine levels in brain glia, including microglia and astrocytes and has protective effects on neuroinflammation-associated neurodegeneration in the brain has not been studied. Here, we found that regorafenib downregulated LPS-induced proinflammatory cytokines levels in BV2 microglia and primary cultured microglia and astrocytes (Figure 1 and Figure 3). A recent study found that the multi-kinase inhibitor sorafenib regulates LPS and PEG-induced anti-inflammatory cytokine IL-10 levels in murine macrophages [26]. In addition, the tyrosine kinase inhibitor dasatinib significantly increases anti-inflammatory cytokine levels in LPS-treated BV2 microglial cells [15]. In this study, we observed that regorafenib selectively upregulated mRNA levels of the anti-inflammatory cytokine IL-4 in BV2 microglial cells (Figure 1). These data suggest that tyrosine kinase inhibitors could differentially affect LPS-induced expression of proinflammatory and anti-inflammatory cytokines in microglia and astrocytes.

Mitogen-activated protein kinase (MAPK) signaling plays an important role in LPS- and/or Aβ-induced proinflammatory cytokine levels. In addition, MAPK signaling is markedly increased in LPS-treated microglial cells and astrocytes in vitro and in vivo (i.e., ERK, AKT) [27]. Importantly, several tyrosine kinase inhibitors alter LPS and Aβ-mediated MAPK signaling to modulate neuroinflammatory responses. For instance, we recently found that dasatinib decreases LPS-mediated AKT phosphorylation to suppress proinflammatory cytokine expression in BV2 microglial cells [15]. Inhibitory effects of ibrutinib and sorafenib on LPS-induced p-AKT signaling have also been reported in BV2 microglial cells and human neuroblastoma cells, respectively [28]. In this study, we found that regorafenib suppressed LPS-induced p-AKT in BV2 microglial cells, primary microglial cells, and primary astrocytes (Figure 1 and Figure 3). These data indicate that these tyrosine kinase inhibitors modulate LPS-induced AKT signaling to affect LPS-induced proinflammatory responses.

The transcription factor STAT3 is highly associated with LPS-induced inflammatory responses [29]. For example, the tyrosine kinase inhibitor BCR-ABL can regulate LPS-induced NF-kB and STAT3 levels in human colorectal cancer cells [30]. In addition, we recently reported that both dasatinib and ibrutinib significantly reduce the LPS-induced increase in nuclear levels of p-STAT3 to regulate LPS-evoked proinflammatory cytokine expression in BV2 microglial cells [15,21]. Here, we observed that LPS-stimulated nuclear STAT3 phosphorylation was also altered in regorafenib-treated BV2 microglial cells (Figure 2), suggesting that modulation of STAT3 signaling is a useful drug target to regulate neuroinflammation and neuroinflammation-related diseases.

Our previous studies demonstrated that the tyrosine kinase inhibitors dasatinib and ibrutinib regulate LPS-induced microglial and astrocyte activation and the expression of proinflammatory cytokines in wild-type mice [15,21]. In the current study, we found that regorafenib decreased LPS-mediated microglial activation and COX-2 levels in wild-type mice, with smaller effects on astrocyte activation (Figure 4 and Figure 5). Based on our findings, the duration of treatment of 5x FAD mice with regorafenib may not have been sufficient to greatly modulate LPS-evoked gliosis in the brain; therefore, longer treatment times should be used in future assessments of the effects of regorafenib on LPS-induced astrocyte activation in wild-type mice. Taken together, our results suggest that tyrosine kinase inhibitors, including regorafenib, could modulate LPS-induced neuroinflammatory responses in vivo.

Chronic neuroinflammation is directly and indirectly associated with learning and memory and synaptic function [31]. Since tyrosine kinase inhibitors and multi-target kinase inhibitors positively regulate LPS-induced neuroinflammatory responses, we hypothesized that tyrosine kinase inhibitors could regulate learning and memory by altering the dendritic spine number, which is associated with cognitive function [15,21]. Indeed, Echeverria et al. demonstrated that the injection of the tyrosine kinase inhibitor sorafenib in aged APP^swe^ mice (a mouse model of AD) rescues working memory by regulating neuroinflammatory responses [32]. However, several studies have shown that sorafenib negatively affects cognitive function in cancer patients by disrupting metabonomic pathways [33,34]. Based on the literature, it is possible that sorafenib positively regulates synaptic/cognitive function in mouse models of AD and in AD patients but not in patients with cancer. However, whether other tyrosine kinase inhibitors could affect cognitive/synaptic function is not well-studied. Therefore, we investigated whether regorafenib modulates dendritic spine formation and found that regorafenib significantly promoted dendritic spine numbers in primary hippocampal neurons and a mouse model of AD (Figure 6), suggesting that regorafenib could positively regulate synaptic/cognitive function in a mouse model of AD. To address this, future studies will determine how regorafenib promotes synaptic/cognitive function and its mechanisms of action in mouse models of AD.

The AD brain is marked by two neuropathological characteristics—Aβ accumulation and tau tangle formation. Several studies have shown an association between tyrosine kinase inhibitors and AD pathology [20,35]. For instance, in Aβ-treated human cells or rat brain cortical cultures, tyrosine phosphorylation of numerous neuronal proteins is increased, including tau, and this tyrosine phosphorylation is inhibited by treatment with the tyrosine kinase inhibitor PP2 [36]. Another study found that genistein, a protein tyrosine kinase (PTK) inhibitor, reduces Aβ production in the rat hippocampus [37]. By contrast, Aβ levels are not altered in dasatinib-injected APP/PS1 mice compared with vehicle-injected APP/PS1 mice [20]. However, whether the multi-target kinase inhibitor regorafenib and other tyrosine kinase inhibitors affect Aβ pathology is not well understood. Thus, we tested the effects of regorafenib on Aβ plaque loads and its mechanisms of action. Importantly, we found that regorafenib significantly reduced Aβ plaque levels in a mouse model of AD (Figure 7). In addition, we examined the molecular mechanisms by which regorafenib alters Aβ pathology and found that regorafenib decreased Aβ plaque loads by altering the levels of BACE1 and the autophagy protein ATG12, which are associated with Aβ (Figure 7) [38,39]. Of course, it is possible that regorafenib affects other Aβ production/degradation-related protein/enzymes to alter Aβ plaque loads. Taken together, our results indicate that regorafenib affects Aβ pathology via a multi-directional pathway.

A recent study reported that the tyrosine kinase inhibitor sunitinib downregulates the tau kinase CDK5 and tau phosphorylation in gp120 Tg mice [40]. In addition, genistein modulates Aβ-induced tau phosphorylation in SH-SY5Y cells [35]. In the present study, we found that regorafenib injection selectively regulated tau phosphorylation in 5x FAD mice by decreasing the activity of the tau kinase GSK3β (Figure 8). Future studies will extend our findings by determining how regorafenib affects both Aβ plaque levels and tau pathology. In conclusion, our results reveal novel functions of regorafenib in the regulation of neuroinflammation and AD-associated pathology, and suggest potential therapeutic effects of regorafenib for AD and other neurodegenerative diseases.

## Supplementary Materials

The following are available online at https://www.mdpi.com/2073-4409/9/7/1655/s1. Figure S1: Regorafenib significantly decreases astrocyte activation in the cortex in 5x FAD mice, Figure S2: Regorafenib does not alter α-secretase ADAM17 levels in the brain in 5x FAD mice. Figure S3: Regorafenib does not alter levels of the Aβ degradation enzyme NEP in the brain in 5x FAD mice, Figure S4: Regorafenib does not affect levels of the autophagy-related protein ATG5 in the brain in 5x FAD mice, Figure S5: Regorafenib does not alter phosphorylation of tau at S202, T205 and T231 in the brain in 5x FAD mice, Figure S6: Regorafenib significantly reduces Tau-5 levels in the cortex in 5x FAD mice.
