# Supplementary material for: Regorafenib Regulates AD Pathology, Neuroinflammation, and Dendritic Spinogenesis in Cells and a Mouse Model of AD
Source: Cells. 2020 Jul 9;9(7):1655. doi: 10.3390/cells9071655 (PMC7408082; doi:10.3390/cells9071655)
Supplement: Supplementary file 1 [file cells-09-01655-s001.pdf]

Regorafenib Regulates AD Pathology, Neuroinflammation, and Dendritic Spinogenesis in Cells and a Mouse Model of AD

Kyung-Min Han [1,2,†], Ri Jin Kang [1,3,†], Hyongjun Jeon [1,+], Hyun-ju Lee [1], Ji-Soo Lee [1], HyunHee Park [1], Seong Gak Jeon [1], Kyoungho Suk [3], Jinsoo Seo [2,*] and Hyang-Sook Hoe [1,2,*]

[1] Department of Neural Development and Disease, Korea Brain Research Institute (KBRI), 61, Cheomdan-ro, Dong-gu, Daegu 41068, Korea

[2] Department of Brain and Cognitive Sciences, Daegu Gyeongbuk Institute of Science & Technology, Daegu 42988, Korea

[3] Department of Pharmacology, Brain Science & Engineering Institute, School of Medicine, Kyungpook National University, Daegu 41944, Korea

* Correspondence: sookhoe72@kbri.re.kr (H.-S.H.); jsseo@dgist.ac.kr (J.S.); Tel.: , +82-53-980-8310 (H.-S.H.); +82-53-420-4835 (J.S.)

† These authors equally contributed to this work.

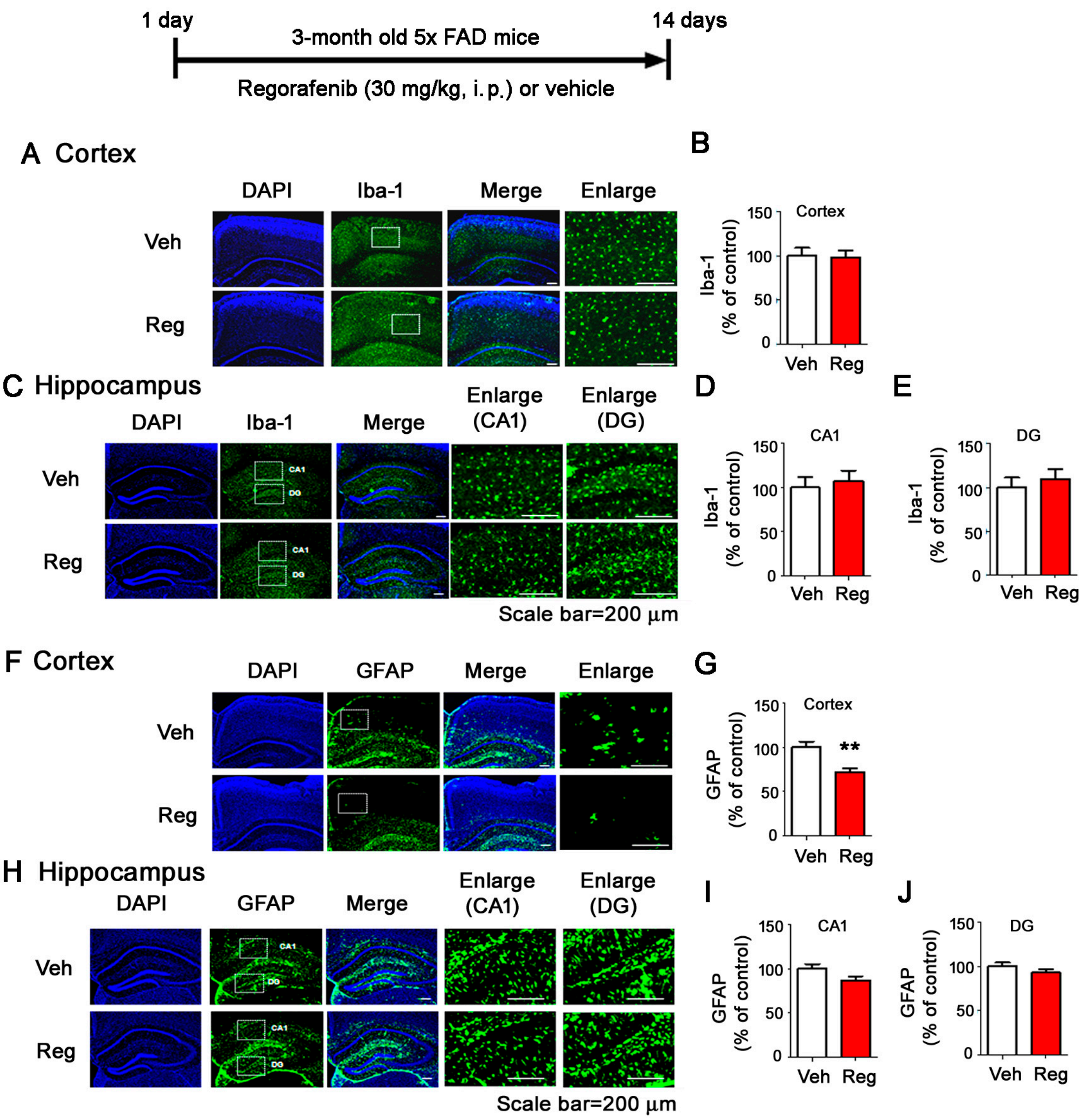

Supplementary Figure S1. Regorafenib significantly decreases astrocyte activation in the cortex in 5x FAD mice. (**A**-**J**) 3-month-old 5x FAD mice were injected with regorafenib (30 mg/kg, i.p.) or vehicle (2% DMSO + 30% PEG + 5% Tween80) daily for 2 weeks, and immunohistochemistry was performed with anti-Iba-1 or anti-GFAP antibodies. Representative images of the cortex (**A**, **F**) and hippocampus (**C**, **H**) are shown. (**B**, **D**, **E**) Quantification of data from **A** (cortex: vehicle, n=4 mice; regorafenib, n=4 mice) and **C** (CA and DG: vehicle, n=4 mice; regorafenib, n=4 mice). (**G**, **I**, **J**) Quantification of data from **F** (cortex: vehicle, n=4 mice; regorafenib, n=4 mice) and **H** (CA and DG: vehicle, n=4 mice; regorafenib, n=4 mice). Data are mean ± SEM, two-tailed Welch's adjusted t-test. ***p*<0.01.

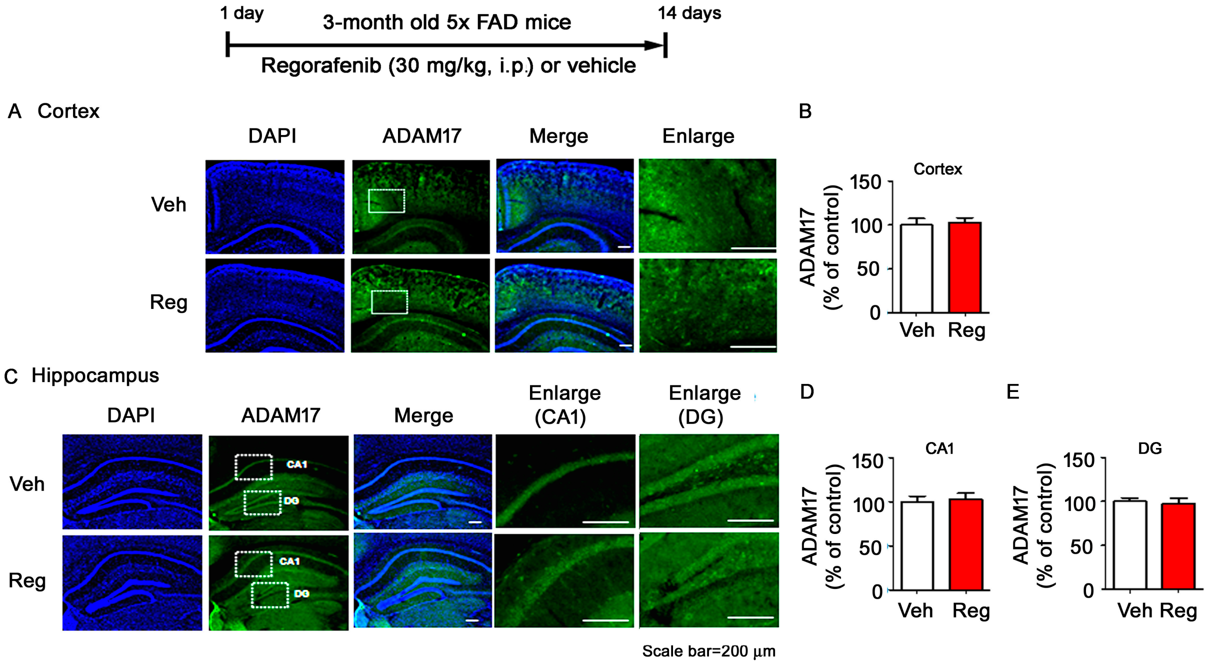

Supplementary Figure S2. Regorafenib does not alter -secretase ADAM17 levels in the brain in 5x FAD mice. (**A**-**E**) 3-month-old 5x FAD mice were injected with regorafenib (30 mg/kg, i.p.) or vehicle (2% DMSO + 30% PEG + 5% Tween80) daily for 2 weeks, and immunohistochemistry was performed with an anti-ADAM17 antibody. Representative images of the cortex (**A**) and hippocampus (**C**) are shown. (**B**, **D**, **E**) Quantification of data from **A** (cortex: vehicle, n=4 mice; regorafenib, n=4 mice) and **C** (CA and DG: vehicle, n=4 mice; regorafenib, n=4 mice). Data are mean ± SEM, two-tailed Welch's adjusted t-test.

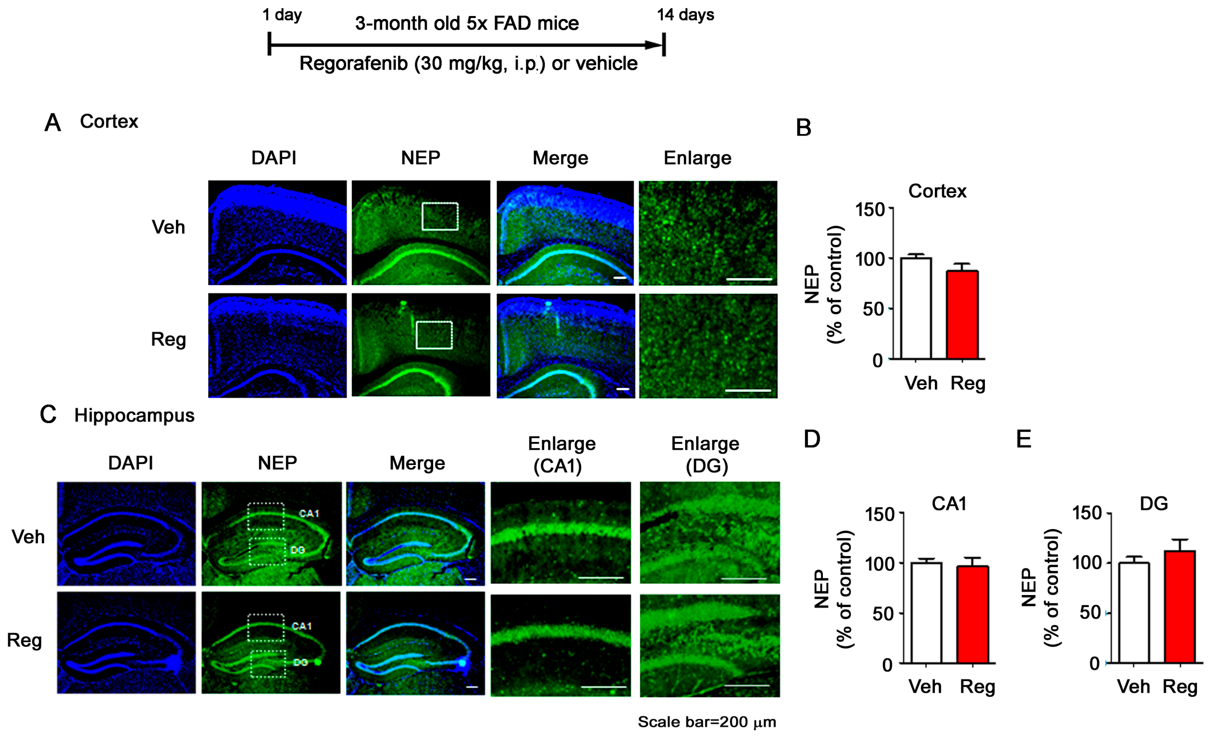

Supplementary Figure S3. Regorafenib does not alter levels of the A degradation enzyme NEP in the brain in 5x FAD mice. (**A**-**E**) 3-month-old 5x FAD mice were injected with regorafenib (30 mg/kg, i.p.) or vehicle (2% DMSO + 30% PEG + 5% Tween80) daily for 2 weeks, and immunohistochemistry was performed with an anti-NEP antibody. Representative images of the cortex (**A**) and hippocampus (**C**) are shown. (**B**, **D**, **E**) Quantification of data from **A** (cortex: vehicle, n=5 mice; regorafenib, n=5 mice) and **C** (CA and DG: vehicle, n=5 mice; regorafenib, n=5 mice). Data are mean ± SEM, two-tailed Welch's adjusted t-test.

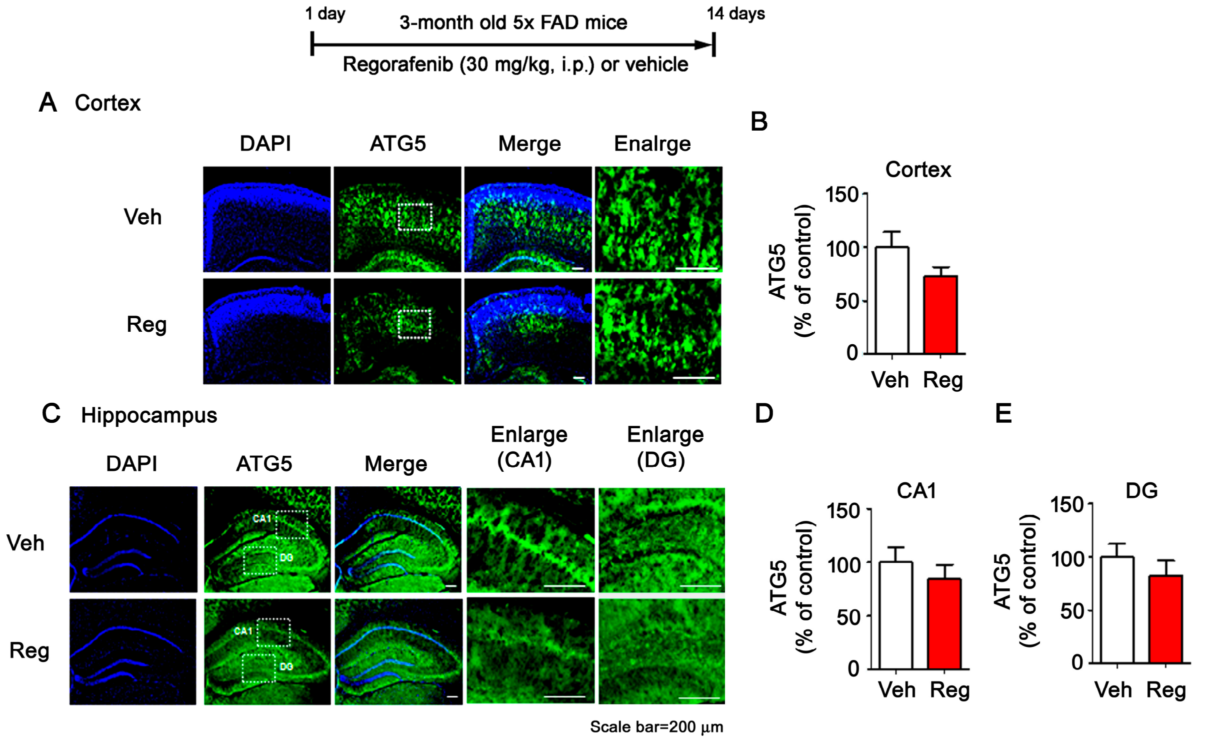

Supplementary Figure S4. Regorafenib does not affect levels of the autophagy-related protein ATG5 in the brain in 5x FAD mice. (**A-E**) 3-month-old 5x FAD mice were injected with regorafenib (30 mg/kg, i.p.) or vehicle (2% DMSO + 30% PEG + 5% Tween80) daily for 2 weeks, and immunohistochemistry was performed with an anti-ATG5 antibody. Representative images of the cortex (**A**) and hippocampus (**C**) are shown. (**B**, **D**, **E**) Quantification of data from **A** (cortex: vehicle, n=4 mice; regorafenib, n=4 mice) and **C** (CA and DG: vehicle, n=4 mice; regorafenib, n=4 mice). Data are mean ± SEM, two-tailed Welch's adjusted t-test.

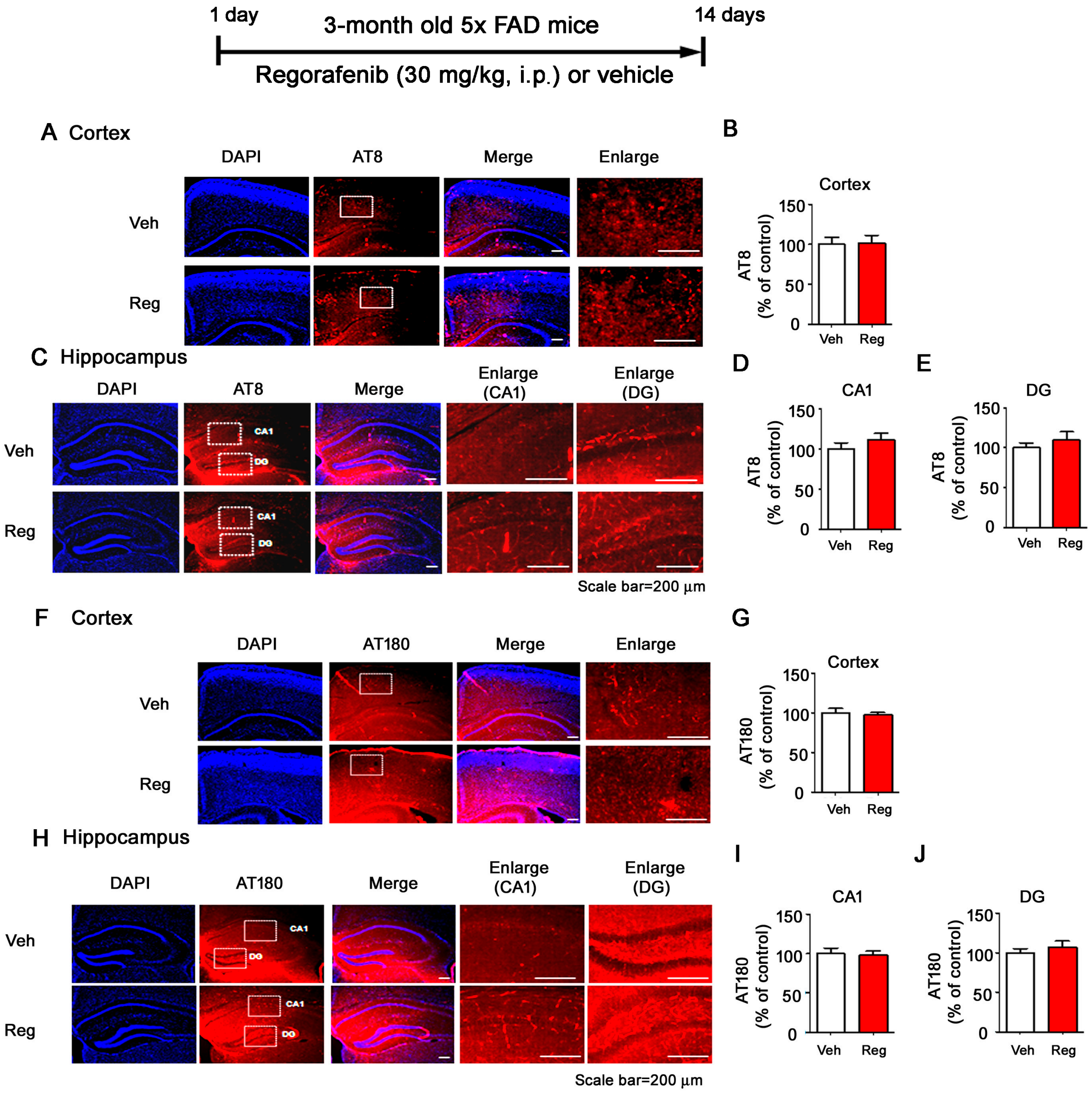

Supplementary Figure S5. Regorafenib does not alter phosphorylation of tau at S202, T205 and T231 in the brain in 5x FAD mice. (**A-J**) 3-month-old 5x FAD mice were injected with regorafenib (30 mg/kg, i.p.) or vehicle (2% DMSO + 30% PEG + 5% Tween80) daily for 2 weeks, and immunohistochemistry was performed with anti-AT8 or anti-AT180 antibodies. Representative images of the cortex (**A**, **F**) and hippocampus (**C**, **H**) are shown. (**B**, **D**, **E**) Quantification of data from **A** (cortex: vehicle, n=4 mice; regorafenib, n=4 mice) and **C** (CA and DG: vehicle, n=4 mice; regorafenib, n=4 mice). (**G**, **I**, **J**) Quantification of data from **F** (cortex: vehicle, n=4 mice; regorafenib, n=4 mice) and **H** (CA and DG: vehicle, n=4 mice; regorafenib, n=4 mice). Data are mean ± SEM, two-tailed Welch's adjusted t-test.

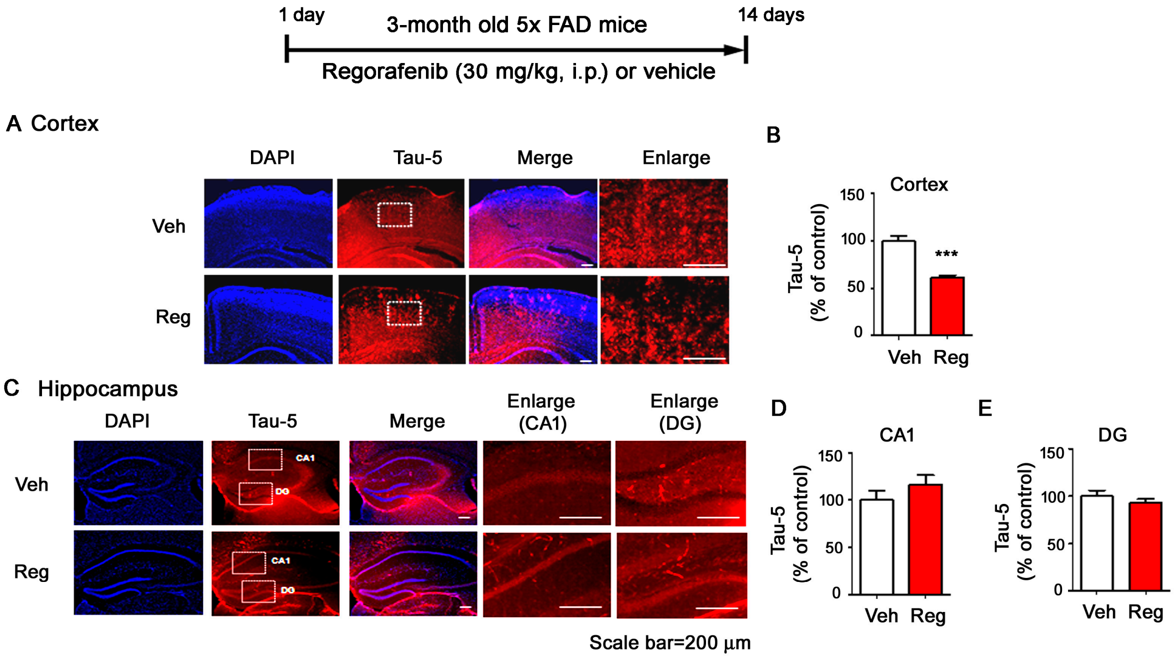

Supplementary Figure S6. Regorafenib significantly reduces Tau-5 levels in the cortex in 5x FAD mice. (**A**-**E**) 3-month-old 5x FAD mice were injected with regorafenib (30 mg/kg, i.p.) or vehicle (2% DMSO + 30% PEG + 5% Tween80) daily for 2 weeks, and immunohistochemistry was performed with an anti-Tau-5 antibody. Representative images of the cortex (**A**) and hippocampus (**C**) are shown. (**B**, **D**, **E**) Quantification of data from **A** (cortex: vehicle, n=3 mice; regorafenib, n=4 mice) and **C** (CA and DG: vehicle, n=3 mice; regorafenib, n=4 mice). Data are mean ± SEM, two-tailed Welch's adjusted t-test. ***p<0.001.
